# Body Composition in Paediatric GLP-1 Receptor Agonist Therapy. Comment on Zaitoon et al. Beyond Weight Loss: Optimizing GLP-1 Receptor Agonist Use in Children. *Children* 2025, *12*, 1427

**DOI:** 10.3390/children13050607

**Published:** 2026-04-28

**Authors:** Nicola Improda, Giada Ballarin

**Affiliations:** Neuroendocrine Diseases and Obesity Unit, Department of Neurosciences, Santobono-Pausilipon Children’s Hospital, Via Egiziaca a Forcella 18, 80139 Naples, Italy; g.ballarin@santobonopausilipon.it

Dear Editor,

We read with great interest the manuscript entitled “Beyond Weight Loss: Optimizing GLP-1 Receptor Agonist Use in Children” by Zaitoon et al. [1]. The authors provide a timely and comprehensive overview of a rapidly evolving field; however, we would like to offer a specific perspective concerning body composition data that warrants further clarification.

The manuscript summarises evidence from the STEP 1 and SURMOUNT-1 trials, noting that Dual-Energy X-ray Absorptiometry (DEXA)-based assessments of the Glucagon-Like Peptide-1 Receptor Agonist (GLP-1RA) semaglutide and the dual Glucose-dependent Insulinotropic Polypeptide (GIP)/GLP-1RA tirzepatide in adult populations show that fat-free mass (FFM) loss accounts for a significant portion (20–38%) of the total weight reduction. Based on this evidence, the authors suggest that it is plausible to expect that the weight loss patterns in paediatric patients might mirror those seen in adults, resulting in a reduction in both fat mass (FM) and FFM during treatment [1].

In our opinion, however, caution is needed before directly extrapolating this phenomenon to children or adolescents. In fact, hormonal changes during growth, especially puberty, drive a steady rise in FFM, muscle mass, and body cell mass [2,3,4,5], which could potentially counteract the deficits in muscle quality and strength reported in adult populations treated with GLP-1RAs to date.

In keeping with this, Apperley et al. assessed body composition through Bioelectrical Impedance Analysis (BIA) in adolescents on liraglutide and observed a significant decrease in fat mass (–4.00 kg; *p* = 0.011) alongside a numerical increase in fat-free mass (FFM) (+1.56 kg; *p* = 0.260) over 3 months [6]. Nevertheless, these preliminary findings regarding FFM preservation warrant caution due to the small sample size (*n* = 16), short duration and retrospective nature of the study [6]. Data from bariatric surgery, which induces hormonal shifts partially mimicking GLP-1RA therapy, show that even non-malabsorptive procedures like sleeve gastrectomy result in an FFM reduction during the first year. However, this loss appears to be transient, as physiological growth drivers in adolescents likely promote a subsequent recovery of lean mass over time [7]. Furthermore, beyond age-related factors, sex-based differences may also influence outcomes, as boys may potentially show more pronounced FM reductions than girls [7]. Definitive evidence is expected from ongoing trials like RESETTLE, which use DEXA and Magnetic Resonance Imaging to precisely assess the long-term impact of GLP-1RAs on weight loss quality and muscle maintenance in youth [8].

Future research on body composition in paediatric GLP-1RA users must address several methodological pitfalls. Regarding DEXA, while it remains the gold standard, its application in large-scale longitudinal paediatric research is hampered by practical challenges such as poor compliance, movement artifacts, and an inability to assess hydration status or cell quality. In contrast, BIA offers distinct practical advantages, emerging as a non-invasive, accessible alternative that facilitates the assessment of both body composition and fluid balance, particularly when longitudinal monitoring is required [6,9,10]. However, the clinical utility of BIA is hindered by significant limitations. First, a lack of inter-device consistency undermines data comparability across multicentric studies [11,12]. Second, there is a scarcity of validated prediction formulas and reference values for paediatric populations, particularly for children with obesity or trained youths [10,11]. Third, BIA accuracy is highly sensitive to hydration fluctuations—a critical confounder during the initial phases of GLP-1RA therapy due to gastrointestinal side effects. Finally, the ethical and logistical challenges of recruiting paediatric control groups in real-world settings continue to hamper the robustness of available data.

Current gaps in our understanding of how GLP-1RAs affect paediatric body composition significantly hinder the development of specific evidence-based clinical recommendations regarding supportive care. Specifically, body composition data are needed to define optimal macro- and micronutrient intake (e.g., targeted protein) and physical exercise regimens (e.g., resistance training) to be prescribed alongside pharmacological treatment.

Therefore, given the rapidly growing and widespread use of GLP-1RAs in paediatric practice, we believe that addressing these complexities in further studies is no longer elective but imperative.
